# Complete Genome Sequence of Vulcanisaeta souniana Strain IC-059, a Hyperthermophilic Archaeon Isolated from Hot Spring Water in Japan

**DOI:** 10.1128/mra.01080-22

**Published:** 2023-01-04

**Authors:** Shingo Kato, Takashi Itoh, Linhuan Wu, Juncai Ma, Moriya Ohkuma

**Affiliations:** a Japan Collection of Microorganisms, RIKEN BioResource Research Center, Tsukuba, Japan; b World Data Center for Microorganisms, Institute of Microbiology, Chinese Academy of Sciences, Beijing, China; Portland State University

## Abstract

Vulcanisaeta souniana strain IC-059^T^ (=JCM 11219^T^) is an anaerobic hyperthermophilic archaeon isolated from a Japanese hot spring field. Here, we report the complete genome sequence (2.43 Mbp) of this strain using a hybrid approach with Illumina short-read sequencing and Nanopore long-read sequencing.

## ANNOUNCEMENT

Vulcanisaeta souniana strain IC-059^T^ (=JCM 11219^T^) is an anaerobic hyperthermophilic archaeon belonging to the phylum *Crenarchaeota* (or *Thermoproteota*), which was isolated from a hot spring field in Sounzan, Hakone, Japan (1). Draft genome sequences of *V. souniana*, determined by short-read sequencing using the Ion Torrent PGM (GenBank accession no. BBBK01000000) and Illumina HiSeq X Ten (GenBank accession no. BMNM00000000) platforms, have appeared in the public databases. However, no complete genome of this species has been reported so far.

*V. souniana* IC-059^T^, which was preserved as frozen stocks in our laboratory, was used in this study. For Illumina short-read sequencing, cells in total 12-L cultures (JCM medium no. 283, 80°C, 13 days) were harvested at 10,000 × *g* for 20 min. Genomic DNA was extracted from the harvested cells by chloroform/phenol extraction and purified by ethanol precipitation as previously described (1). The purified DNA was sent to BGI Genomics (Yantian District, Shenzhen, China) for short-read sequencing using the Illumina HiSeq X Ten platform (150-bp paired-end format) as the Global Catalogue of Microorganisms (GCM) 10K prokaryotic type strain sequencing project (2, 3). Briefly, the DNA was sheared into smaller fragments using the Covaris S/E210 ultrasonicator, and the ends were blunted using T4 DNA polymerase, the Klenow fragment, and T4 polynucleotide kinase. After adding an A base to the 3′ end of the blunt phosphorylated DNA fragments, adapters were ligated to the ends of the DNA fragments. Index tags were introduced into the adapters by PCR. The obtained library was used for sequencing. BGI provided a total of 3,730,776 high-quality paired-end short reads (559,616,400 bp). We checked the quality of the short reads using FastQC (https://github.com/s-andrews/FastQC). It should be noted that the same short reads were used to construct the draft genome sequence (GenBank accession no. BMNM00000000). For Nanopore long-read sequencing, genomic DNA was newly prepared from cells in total 2 L of medium (JCM medium no. 297) at 80°C for 8 days. DNA extraction and purification were performed as described above. The purified DNA, without size selection, was used for library construction with a rapid sequencing kit (SQK-RAD004; Oxford Nanopore Technologies [ONT], UK). The library was sequenced using a Nanopore MinION instrument with a FLO-MIN106 (R9.4.1) flow cell. Base calling of the raw data was performed using Guppy version 2.1.3 (ONT). High-quality (quality score, ≥8) and long (≥2,000 bp) reads were extracted using NanoFilt version 2.2.0 (4) with the “headcrop” option set to 100 bp. This resulted in 6,084 reads (30,339,403 bp) with an *N*_50_ value of 5,669 bp. *De novo* assembly of the genome using both these short and long reads was performed using Unicycler version 0.4.7 (5). Genome annotation was performed using DFAST version 1.6.0 (6), including Prodigal (7) for gene prediction, Barrnap (https://github.com/tseemann/barrnap) for predicting the rRNAs, tRNAscan-SE (Archaea) (8) for predicting the tRNAs, and CRT (9) for determining the CRISPR regions. For the above analysis, default parameters were used except where otherwise noted.

The hybrid assembly resulted in a circular contig of 2,432,148 bp with a G+C content of 45.1% and 453-fold average genome coverage. The genome contained 2,664 protein-coding regions (CDSs), 1 each of 16S and 23S rRNA genes, and 46 tRNA genes. In addition, all the key genes for sulfate reduction, i.e., *hppA*/*ppaC*, *dsrABCMK*, *aprAB*, *sat*, and *qmoABC* (10), were found in its genome. Although the possible use of sulfate as an electron acceptor by *V. souniana* has been reported (1), its capability for sulfate reduction is still controversial (10). Further cultivation experiments are needed to clarify this point.

### Data availability.

The complete genome sequence determined in this study has been deposited at DDBJ/ENA/GenBank under the accession no. AP026830. The raw data are available under the DRA accession no. DRA014838.
